# Microbiomes associated with infective stages of root-knot and lesion nematodes in soil

**DOI:** 10.1371/journal.pone.0177145

**Published:** 2017-05-04

**Authors:** Ahmed Elhady, Ariadna Giné, Olivera Topalovic, Samuel Jacquiod, Søren J. Sørensen, Francisco Javier Sorribas, Holger Heuer

**Affiliations:** 1 Dept. Epidemiology and Pathogen Diagnostics, Julius Kühn-Institut—Federal Research Centre for Cultivated Plants, Braunschweig, Germany; 2 Department of Plant Protection, Faculty of Agriculture, Benha University, Benha, Egypt; 3 Departament d’Enginyeria Agroalimentària i Biotecnologia, Universitat Politècnica de Catalunya, Castelldefels, Spain; 4 Section of Microbiology, Department of Biology, University of Copenhagen, Copenhagen, Denmark; INRA, FRANCE

## Abstract

Endoparasitic root-knot (*Meloidogyne* spp.) and lesion (*Pratylenchus* spp.) nematodes cause considerable damage in agriculture. Before they invade roots to complete their life cycle, soil microbes can attach to their cuticle or surface coat and antagonize the nematode directly or by induction of host plant defenses. We investigated whether the nematode-associated microbiome in soil differs between infective stages of *Meloidogyne incognita* and *Pratylenchus penetrans*, and whether it is affected by variation in the composition of microbial communities among soils. Nematodes were incubated in suspensions of five organically and two integrated horticultural production soils, recovered by sieving and analyzed for attached bacteria and fungi after washing off loosely adhering microbes. Significant effects of the soil type and nematode species on nematode-associated fungi and bacteria were revealed as analyzed by community profiling using denaturing gradient gel electrophoresis. Attached microbes represented a small specific subset of the soil microbiome. Two organic soils had very similar bacterial and fungal community profiles, but one of them was strongly suppressive towards root-knot nematodes. They were selected for deep amplicon sequencing of bacterial 16S rRNA genes and fungal ITS. Significant differences among the microbiomes associated with the two species in both soils suggested specific surface epitopes. Among the 28 detected bacterial classes, Betaproteobacteria, Bacilli and Actinobacteria were the most abundant. The most frequently detected fungal genera were *Malassezia*, *Aspergillus* and *Cladosporium*. Attached microbiomes did not statistically differ between these two soils. However, *Malassezia globosa* and four fungal species of the family Plectosphaerellaceae, and the bacterium *Neorhizobium galegae* were strongly enriched on *M*. *incognita* in the suppressive soil. In conclusion, the highly specific attachment of microbes to infective stages of phytonematodes in soil suggested an ecological role of this association and might be involved in soil suppressiveness towards them.

## Introduction

Nematodes are exposed to a myriad of microorganisms in the soil. Among the most devastating plant-parasitic groups are root-knot (*Meloidogyne* spp.) and root-lesion nematodes (*Pratylenchus* spp.). *Meloidogyne* spp. are sedentary endoparasites characterized by infective second-stage juveniles (J2) that enter the host roots and form multinucleate feeding sites called giant cells. As a consequence the root galls are formed through which swollen females protrude and deposit egg masses usually on the gall surface. *Pratylenchus* spp. are migratory endoparasitic nematodes that cause the formation of root lesions as all infective stages move and feed inside the roots. Until reaching the plant, J2 of *Meloidogyne* and all life stages of *Pratylenchus* migrate through the soil. Many attempts have been engaged to successfully control plant-parasitic nematodes. This includes the biological control by soil-borne microorganisms. The inability of pathogens to survive and establish, or the ability to establish but not to cause a disease to an important extent in different soils is attributed to soil suppressiveness. Biotic suppressiveness of soils may be general, where complex ecological inter-actions suppress several pathogens, or specific, where one or a few species antagonize a particular pathogen [1]. A soil suppressive to *Pratylenchus thornei* was reported, albeit no microorganisms contributing to the suppressiveness were identified [2]. Egg parasites of phytonematodes acting against cyst-forming nematodes [3, 4] or *Meloidogyne* [5–9] have been suggested to contribute to soil suppressiveness. However, the correlation between the abundance of egg parasites and soil suppressiveness often is rather weak [9, 10].

The most studied case of a microbial antagonism to plant-parasitic nematodes involved the bacterium *Pasteuria penetrans*. Its endospores were found in empty females of *Meloidogyne javanica* in suppressive vineyard soils in South Australia [11]. The initial and most important step in this host-parasite interaction is the attachment of endospores to the cuticle of J2 of *M*. *javanica* in the soil before entering roots [12]. Cuticles of plant-parasitic nematodes are covered with a highly glycosylated surface coat which is secreted from the hypodermis. The surface coat is very important in plant-nematode interaction as its molecules are among the first to interface with the plant defense system [13], and attached microbes may interfere with the nematode-plant interaction. Carbohydrate-recognition-domains of the surface coat specifically binding parasitic or predatory soil microorganisms have been proposed [14], and differences in the binding properties of the surface coat among nematode species were shown [15]. Recently, specific fungal and bacterial species were found to be associated with the cuticle of J2 of *Meloidogyne hapla* in soil that suppressed the nematode fecundity on tomato [16]. However, microbiomes associated with infective stages of different nematode species in soil and the influence of soil microbial communities on these microbiomes were barely studied.

The objective of this study was to identify the bacteria and fungi that attach to the infective stages of the nematode species *Meloidogyne incognita* and *Pratylenchus penetrans* in different soils. We hypothesized that a specific subset of soil microbes binds to the nematodes. As epitopes on the surface of the two nematodes may be specific for each species it was tested whether the sets of attached bacteria and fungi differ significantly between the two nematode species in the same soil. The nematode-associated microbiomes were investigated in seven soils with varying suppressiveness towards *M*. *incognita* in horticulture to assess the influence of the soil microbial community to which the nematode is exposed to. The study aimed to provide a solid base for investigating the ecological role of microbes associated with infective stages of plant-parasitic nematodes in soil, especially with respect to soil suppressiveness and plant protection.

## Materials and methods

### Experimental design, nematodes and soils

*Pratylenchus penetrans* and *M*. *incognita* were cultured to obtain large numbers of inoculum for soil incubation. Prior to propagation on discs of surface sterilized carrots *P*. *penetrans* was disinfected with 0.02% HgCl_2_ for 3 minutes followed by a treatment with streptomycin sulfate (4000 ppm) for 3 minutes. Two to four months old carrot discs were aseptically cut into very small pieces and extracted on a sterilized Baermann funnel. *M*. *incognita* was reared on susceptible tomato seedlings cultivar Moneymaker for 8 weeks in greenhouse under 16: 8 h photoperiod at 26°C. Egg masses were picked off from infected roots (provided by J. Hallmann, Münster, Germany), treated with 0.5% NaOCl for 30s, and hatched in sterile tap water at room temperature. Both nematodes were washed with sterile water on sterile 20 μm sieves (CellTrics® filters, Sysmex, Norderstedt, Germany) before incubation in soil suspensions overnight. Seven commercial horticulture production sites conducted under organic (five sites) or integrated (two sites) production standards of Catalonia (northeastern Spain) were selected based on their observed field suppressiveness [9] or different levels of fungal egg parasitism of root-knot nematodes [10]. Four out of seven soils were located in the same cropping area with similar agro-climatic conditions, M10.23, 10.41, 10.55, and 10.56. In soil M10.23, the *Meloidogyne* population declined in the rotation from zucchini, tomato, to radish / spinach until egg masses were not detected anymore [10]. Such suppressiveness was not observed in M10.56 soil. The fertilization in sites conducted under organic production, M10.16, M10.23, M10.41, M10.55 and M10.56, were done with composted sheep or cow manure, and in some sites it was combined with chicken manure to achieve a total rate between 1.7 and 2 kg m^-2^. The compost was incorporated into the soil just before transplanting each crop. In addition, the non-commercial plant parts at the end of each crop were incorporated as green manure. The rotation sequences included several plant species belonging to the families Brassicaceae, Chenopodiaceae, Compositae, Cucurbitaceae, Fabaceae, and Solanaceae. In sites under integrated production, M10.43 and M10.45, fertilization was done with humus combined with chemical fertilizers. The rotation sequences included crops belonging to the Cucurbitaceae, Fabaceae, and Solanaceae. The physicochemical properties and enzymatic activity of these soils are shown in S1 Table. Soil samples used in this study were taken in February 2015, just at transplanting the spring-summer crop.

To efficiently disrupt soil aggregates and release microbes into suspension, 5 g of 2-mm sieved soil was mechanically treated in a Stomacher blender (Seward, London, United Kingdom) three times at 60 s and high speed: once with 20 ml sterile deionized water, followed by 15 ml sterile phosphate-buffered saline containing 0.3 g of Chelex-100 (Bio-Rad, Hercules, USA), and finally with 15 ml of autoclaved 0.02% sodium deoxycholate / 0.5% polyethylene glycol [17]. After each blending soil particles were pelleted at 500 x g for 2 min, the supernatant was decanted into a sterile 50 ml tube, and the pellet transferred back into the Stomacher bag. The combined supernatants of each sample were centrifuged for 20 min at 4000 x g at 4°C to pellet microbes. The soil microbial pellet was washed once with sterile tap water to remove chemical residues, resuspended in 40 ml sterile tap water, and sieved through 5 μm sieve to remove larger soil particles. For each soil four replicate 15 ml tubes with 1000 J2 of *M*. *incognita* or mixed stages of *P*. *penetrans* in 5 ml soil suspension were incubated horizontally with gentle shaking overnight (20°C). The nematodes were recovered from soil suspensions on sterile 20 μm sieves (CellTrics) and washed 5 times with 10 ml sterile water. Nematodes were transferred with 0.1 ml sterile water into FastDNA Spin bead beating tubes (MP Biomedicals, Santa Ana, CA) and kept at -20°C until DNA extraction.

### Analysis of microbiomes associated with nematodes

Total DNA from nematodes with adhering microorganisms was extracted using the FastPrep FP120 bead beating system (MP Biomedicals, Santa Ana, CA) for 30s at high speed to efficiently disrupt bacterial and fungal cells, and the FastDNA Spin Kit for soil (MP Biomedicals). Total DNA was extracted from a soil pellet of 1 ml suspension in the same way for comparison of the microbial communities from nematodes to those of the surrounding soil. DNA was further purified using the GENECLEAN Spin Kit (MP Biomedicals).

For microbial community analysis by high-throughput amplicon sequencing, 16S rRNA ribosomal gene or fungal ITS fragments were amplified from total DNA samples by an initial PCR step. Bacterial primers used for this PCR were 341F (5’-CCTAYGGGRBGCASCAG-3‘) and 806R (5’-GGACTACNNGGGTATCTAAT-3‘), which flank the 460bp variable V3-V4 region of the 16S rRNA gene of the target group Prokaryotes including domains of Archaea and Bacteria [18, 19]. Fungal ITS fragments were amplified by PCR with primers gITS7 and ITS4 as described [20]. A second amplification step of the corresponding amplicon using the same primers with attachment of adaptors and barcode tags was done as described [19]. Purification and size-selection (removal of products less than 100bp) of PCR amplicon products was performed using Agencourt AMPure XP beads (Beckman Coulter, Brea, CA, USA) according to manufacturer’s instructions. The concentration of the purified amplicon samples was measured using a Qubit Fluorometer (Life Technologies, Carlsbad, CA, USA), the samples were pooled and adjusted to equimolar concentrations, concentrated using the DNA Clean and Concentrator™-5 kit (Zymo Research, Irvine, CA, USA), and finally subjected to 2x250 bp paired-end high-throughput sequencing on an Illumina® MiSeq® platform (Illumina, San Diego, CA, USA).

For PCR-DGGE community profiling, fungal ITS or bacterial 16S rRNA gene fragments were amplified from total DNA of nematode or soil as previously described [21]. Shortly, bacterial 16S rRNA gene fragments were amplified using the primer pair F984GC / R1378. The fungal ITS fragments were amplified using a nested PCR approach with primer pairs ITS1F / ITS4 and ITS1FGC / ITS2. DGGE was done using the PhorU_2_ system (Ingeny, Goes, The Netherlands) as previously described [21]. To identify the bacterial and fungal species corresponding to some of the bands of nematode associated DGGE profiles, PCR products were cloned using the vector pGEM-T and *Escherichia coli* JM109 high-efficiency competent cells (Promega, Madison, USA). Cloned amplicons corresponding in electrophoretic mobility to nematode-specific bands were sequenced (Macrogen, Amsterdam, The Netherlands).

### Data analysis and statistics

Sequence demultiplexing was done using the MiSeq Controller Software and diversity spacers were trimmed using Biopieces (www.biopieces.org). Sequence mate-pairing and filtering was done using Usearch v7.0.1090 [22].

For bacterial 16S rRNA gene sequences, OTU clustering, dereplication and singleton removal was performed using Uparse [23]. Chimera checking and removal was performed using Usearch and the ChimeraSlayer package [24]. Representative sequences were defined for each OTU with a threshold of 0.8 using Mothur v.1.25.0 [25]. A unifrac phylogenetic tree was built using Greengenes [26] with QIIME wrappers for PyNAST [27], FastTree [28], and alignment filtering [29]. Sequence contingency tables were exported at the species level for bacteria (97% similarity threshold).

Fungal ITS sequences were assigned to the most similar species hypothesis (SH) in the UNITE version 7.0 database [30] using Megablast [31]. If a sequence had the same bit score to more than one SH then it was assigned to the most abundant SH in the dataset. Counts of uniquely assigned sequences were summed up per sample in an OTU abundance table. OTU were discarded when the assigned sequences were less than 95% similar to any SH, or had less than 100 bp alignment length, or had highest similarity to non-fungal ITS.

The multivariate analysis on OTU abundance tables was done as described [32] with the PAST software [33] and the R software version 3.0.2 [34] with the package vegan [35, 36] and ade4 [37]. A constrained ordination method was applied on individual PCA plots from 16S rRNA gene and ITS profiles (also known as BGA: Between Group Analysis) by grouping replicates together. The replicate grouping significance was inferred by a null-model generated with a Monte-Carlo simulation implementing 10,000 group permutations in order to establish the random constrain effect on the PCA inertia, as described previously [32]. Two-way PERMANOVA tests were performed on the Bray-Curtis dissimilarity profiles using 10,000 permutations to decipher the significance and contribution of each tested factors (1: nematode species and 2: soil used). Since the replicate heterogeneity was very important, a complementary SIMPER analysis was performed to extract the contribution of each individual OTU to the Bray-Curtis dissimilarity profiles. Generalized heatmaps were generated using the R package *gplot* to highlight the abundant OTU which were most discriminating between the microbiomes of *M*. *incognita* and *P*. *penetrans*. To assess the numerical importance of bacterial and fungal taxa, log-transformed relative abundances were used (log(N_O,S_/N_S_*1000+1), with N_O,S_: number of sequences of OTU O in sample S, N_S_: number of sequences of sample S). As they displayed the most marked differences, raw fungi OTU count profiles between the two nematode species were analyzed regardless of the soils by means of a negative binomial distribution under generalized linear model fit (nbGLM) with the package EdgeR [38]. Significance between OTU counts was inferred with a likelihood ratio test after post-hoc false discovery rate multiple correction (LHR FDR, P < 0.05).

DGGE gels were analyzed using the software GelCompar II version 6.6 (Applied Maths, Ghent, Belgium) [21]. Lanes were normalized with common bands as internal standard. Pairwise similarities of the DGGE profiles by Pearson correlation were determined, and cluster analysis was done by the unweighted pair group method with arithmetic averages (UPGMA). The similarity matrices were used to test for significant treatment effects by a permutation test [39]. Community differences (D) between treatments were calculated from the average similarity among samples from the same treatment minus the average similarity between samples from different treatments.

DNA sequences were deposited in the NCBI SRA database under PRJNA349130, and at NCBI GenBank under accession numbers KY432379-KY432398.

## Results

### Microbes associated with infective stages of nematodes in seven horticultural soils analyzed by PCR-DGGE

The fungal and bacterial communities associated with *M*. *incognita* or *P*. *penetrans* in seven soils were analyzed by PCR-DGGE profiling of fungal ITS2 fragments and bacterial 16S rRNA genes. The soils were derived from horticulture in Spain and were reported to vary in suppressiveness towards root-knot nematodes, with soil M10.23 being highly suppressive. The microbial communities attached to the nematodes were analyzed before and after incubation in soil suspensions.

The fungal PCR-DGGE profiles of *P*. *penetrans* in the different soils comprised 12 bands on average, while for *M*. *incognita* about 25 bands could be detected in each sample (S1 Fig). The fungal patterns differed significantly between the two nematode species as revealed by a constrained permutation test based on the pairwise Pearson correlations (*P* < 0.001). Thus different fungal species attached to *P*. *penetrans* than to *M*. *incognita* in the same soil suspension. In particular one fungal type (S1 Fig, band no. 2) was highly abundant on *P*. *penetrans*. This band was already present before inoculation of the nematodes in soil. Other fungal bands on *P*. *penetrans* varied between samples and were typically found for several soils. Fungal patterns for *M*. *incognita* varied less, and specific bands were detected after incubation in several soils. Two bands were specific for *M*. *incognita* in two or three of all studied soils, respectively (S1 Fig, bands no. 3 and 5). Several bands were specifically associated with only one soil (S1 Fig, bands no. 6–19). Only a few soils had a significant influence on attached fungi, with soils M10.56 and M10.43 especially differing from others (soils M10.23 vs. M10.43, and M10.56 vs. M10.43 for *M*. *incognita*; soils M10.56 vs. M10.43 and M10.56 vs. M10.45 for *P*. *penetrans*). Overall, many fungal attachers seem to occur as minor populations in diverse soils and show specificity in attachment to one of the nematode species analyzed.

In contrast to the fungal PCR-DGGE patterns, the profiles of the attached bacterial communities did not reveal a statistically significant difference between *M*. *incognita* and *P*. *penetrans* over all soils after incubation in soil suspensions, as analyzed by a global constrained permutation test based on Pearson correlations. This was due to high variation among replicate samples, especially for *P*. *penetrans* (S2 Fig). However, clearly distinct patterns for each nematode species were apparent after incubation in soils M10.55, M10.23 and M10.56, giving evidence that also bacteria attached specifically to one or the other nematode species in a soil suspension. Consequently, some bacteria were only detected on *M*. *incognita* (S2 Fig, band no. 3 assigned to *Burkholderia lata* with 97% sequence identity; band no. 6 in soils M10.23 and M10.56), or only on *P*. *penetrans* (band no. 5). DGGE profiles of the bacterial communities adhering to *M*. *incognita* or *P*. *penetrans* differed significantly among the tested soils (*P* = 0.027 and 0.004, respectively). In pairwise comparisons, bacteria attached to *M*. *incognita* significantly differed between soils M10.16 and M.10.23, and bacteria attached to *P*. *penetrans* differed between soils M10.16-M10.56, M10.16-M10.43, M10.55-M10.56, M10.55-M10.43, and M10.56-M10.45. A few bands were shared among nematodes from all soils (S2 Fig, band no.1 assigned to *Burkholderia* sp. with 95% sequence identity, band no. 2 assigned to *Fusicatenibacter saccharivorans* with 99.5% identity), while other bacteria were specific for one or two soils (S2 Fig, band no. 6 for soil M10.16 assigned to *Burkholderia caledonica* with 98% identity, band no. 8 for soil M10.45 assigned to *Acinetobacter johnsonii* with 98.9% identity). The vast majority of fungi and bacteria associated with the nematodes after incubation in soil were not present before inoculation on the nematodes, and they were not among the dominant microbes in soil, suggesting highly specific microbial recruitment and attachment.

In contrast to the attached fractions, the total fungal and bacterial communities of the analyzed soils differed significantly among each other (S3 and S4 Figs). A notable exception was soil M10.23 and soil M10.56 which were located in the same cropping area with similar agro-climatic conditions. The two soils were most similar in fungal and bacterial community structure, but differed in the microbes that attached to *M*. *incognita*, suggesting that not the dominant microbial populations but rather specifically attaching less abundant microbes interact with these J2 in soil (S1 and S2 Figs). The majority of soils with organic production, except M10.16, were discriminated from those conducted under integrated management by the first order cluster at a similarity score of 46% and 32% for fungal and bacterial communities, respectively (S3 and S4 Figs). In contrast, fingerprints of nematode-associated fungal or bacterial communities did not show such a clustering. Furthermore, the similarity values of DGGE profiles from nematode and corresponding soil samples were not correlated, suggesting distinct fungal and bacterial communities and highly specific attachment of minor populations to the nematodes (r^2^ < 0.001).

### Microbes attached to nematodes in soils M10.23 and M10.56 analyzed by high-throughput amplicon sequencing

The fungal and bacterial communities associated with *M*. *incognita* or *P*. *penetrans* in the similar soils M10.23 and M10.56 differing in suppressiveness towards root-knot nematodes were analyzed by high-throughput sequencing of 16S rRNA genes and fungal ITS2 fragments. From the 18 samples (2 soils x 2 nematodes x 4 replicates, plus two control samples from nematodes prior to incubation), in total 239,000 and 892,000 high quality paired sequences were obtained for bacteria and fungi, respectively. These were assigned to 312 bacterial OTU and 827 fungal SH. Only 68 bacterial and 181 fungal OTU were detected in 4 or more samples. Among the 28 detected bacterial classes, Betaproteobacteria were most prominent, followed by Bacilli, Actinobacteria, Clostridia, Gamma- and Alphaproteobacteria. Bacteroidetes, Proteobacteria, Firmicutes and Actinobacteria were the most important phyla, and Gemmatimonadetes were present in 13 of the samples. The most abundant genera were *Burkholderia*, *Streptococcus*, *Pelomonas*, *Ralstonia*, and *Bacillus*. Only one bacterial OTU was detected in all samples, which was assigned to the order Clostridiales. The most frequently detected fungal genera were *Malassezia* (most abundant species: *M*. *restricta*, *M*. *globosa*), *Aspergillus* (*A*. *niger*), *Cladosporium* (*C*. *exasperatum*), an unidentified Nectriaceae (SH220702.07FU), an unidentified Ascomycota (SH195297.07FU), *Penicillium* (*P*. *aurantiogriseum*, *P*. *chrysogenum*), *Alternaria* (*A*. *alternata*), an unidentified Sordariomycetes (SH190975.07FU), *Cryptococcus* (*C*. *heimaeyensis*, *C*. *fuscescens*), *Stemphylium* (*S*. *herbarum*), *Mortierella*, and *Chaetomium* (*C*. *jatrophae*).

PERMANOVA analysis based on Bray-Curtis dissimilarities of the relative species abundances in the nematode samples revealed highly significant differences of attached bacteria and fungi between the two nematodes (Table 1). The effect of the soil type (M10.23 or M10.56) on microbial attachment to the nematode surface was not statistically significant in this test (Table 1). The significant interaction between the factors nematode and soil for attached bacteria suggested that the specificity of bacterial attachment was not equally strong in both soils. Substantial variation in detected OTU among samples of the same treatment explained the rather low fraction of variance (r^2^) attributed to the nematode. One of the detected bacterial OTU had a sequence that was identical to the 16S rRNA gene of chloroplasts of *Lactuca sativa* or several other plants. It was associated with both nematodes in both soils, but it was more frequent on *M*. *incognita* and not detected on the inoculated *P*. *penetrans*. However, it did not affect the results. The significance of the nematode species effect on the associated bacterial community remained after it was removed (Table 1, *P*−Chloroplast < 0.01).

**Table 1:** Effect of nematode species and soil type on the structure of the bacterial and fungal community attached to the infective stages of the nematodes in soil.

Effect	Bacterial community			Fungal community	
	r^2^	*P*	*P*—Chloroplast	r^2^	*P*
Soil M10.23 vs. M10.56	0.07	0.18	0.15	0.05	0.72
*M*. *incognita* vs. *P*. *penetrans*	0.11	0.003	0.004	0.17	0.001
Nematode species x Soil type	0.11	0.004	0.006	0.06	0.57

PERMANOVA based on Bray-Curtis dissimilarities between samples with 10,000 permutations, with r^2^ reflecting the fraction of variance attributed to the effect, and ***P***−Chloroplast the significance after removal of chloroplast sequences.

**Fig 1:**
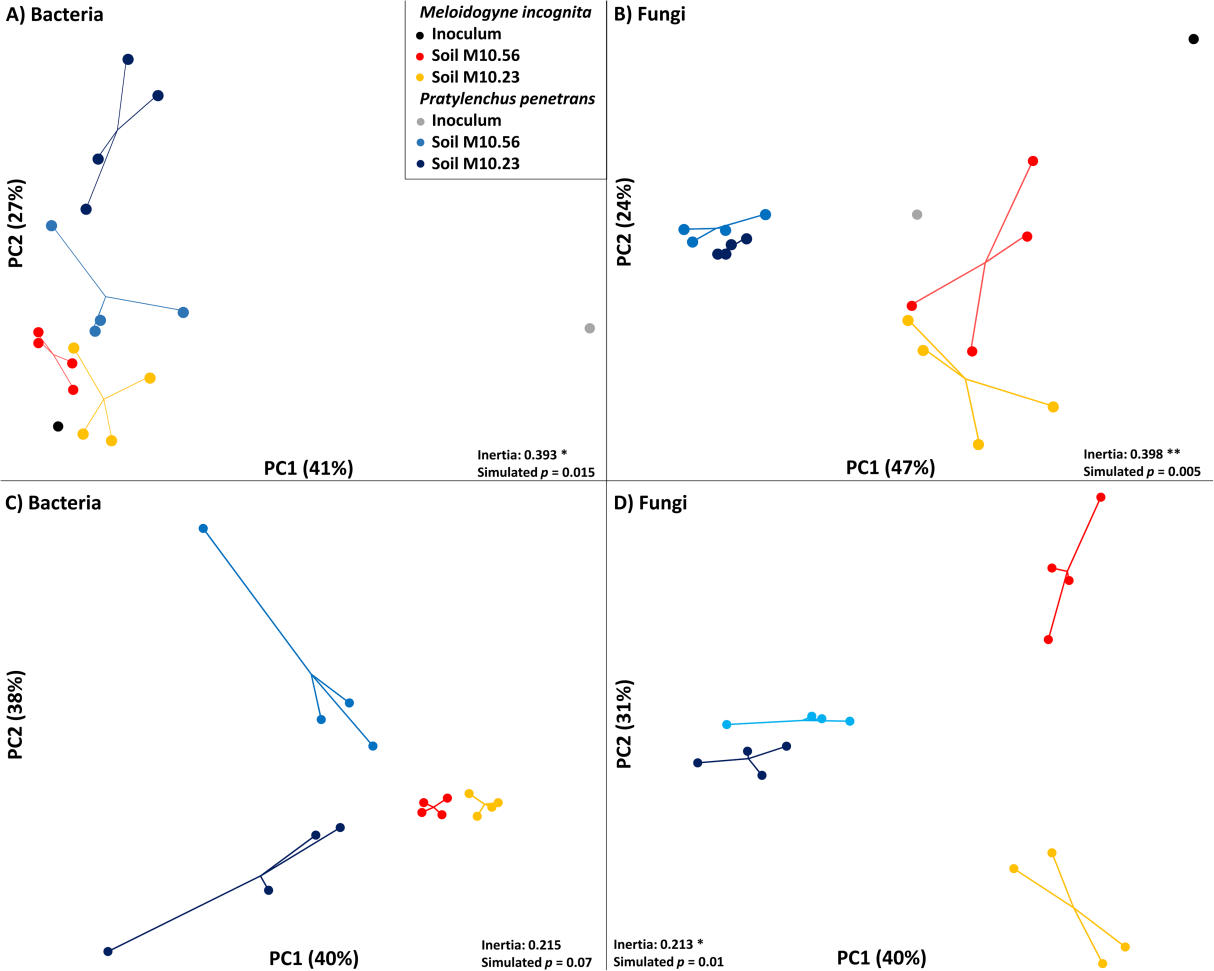
Constrained principle component analysis of bacterial and fungal communities associated with infective stages of nematodes in two soils, and before inoculation. A Between Group Analysis (BGA) was applied by grouping replicate samples together. Significance of the replicate constrain was inferred on the PCA inertia by a Monte-Carlo simulation through 10,000 group permutations (Bacteria *P* = 0.015; Fungi *P* = 0.003). Principal component 1 (PC1) and 2 (PC2) were plotted with (A-B) or without including the microbial community that was found on the inoculated nematodes, and the variance that they explain is shown in parenthesis.

Constrained principal component analysis of log-transformed relative abundances of bacterial and fungal OTU indicated that the microbial communities associated with *M*. *incognita* and *P*. *penetrans* in soil differed (Fig 1). Both profiles revealed a significant, non-random distribution of the samples according to replication, which was more pronounced for Fungi compared to Bacteria (Bacteria *P* = 0.015; Fungi *P* = 0.005). An influence of the incubation in soil M10.23 or M10.56 on attached bacteria and fungi was apparent, although less pronounced than the effect of the nematode species and mainly reflected by PC2. The inoculated nematodes carried also some bacteria and fungi but the incubation in soil clearly determined the microbiomes of the nematodes. The low-diversity microbial communities detected on the nematodes before baiting in soil were highly different from the attached communities after baiting, especially for *Pratylenchus*, thus this variance dominated PC1 (Fig 1A and 1B). Attached bacterial communities varied more for *P*. *penetrans* than for *M*. *incognita* (Fig 1C), and the soil type had a stronger effect which explained the significant interaction between nematode and soil (Table 1). Fungal communities varied much more for *M*. *incognita* than for *P*. *penetrans* as reflected mainly by PC2 (Fig 1D).

**Fig 2:**
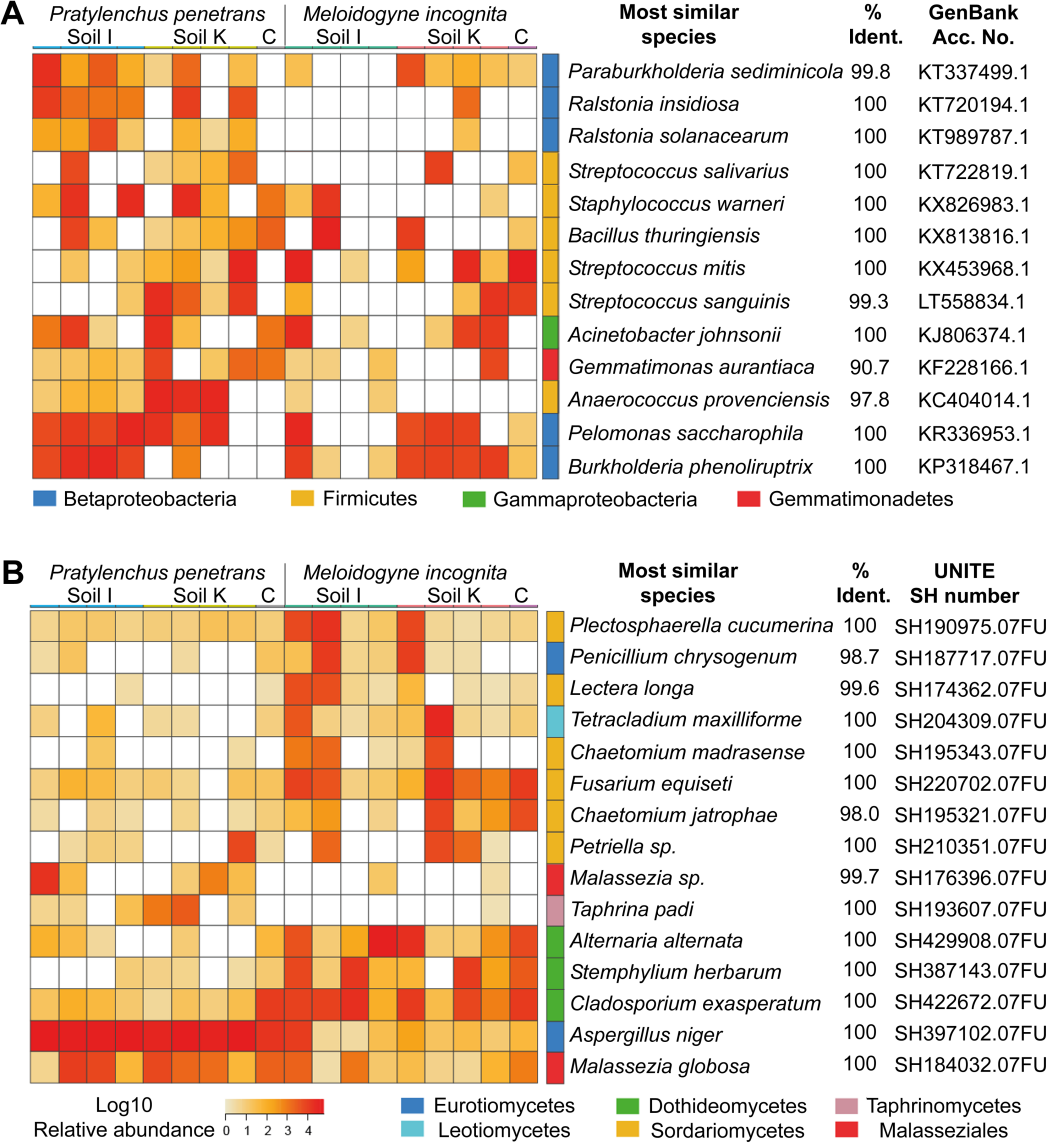
**Heatmaps of the OTU that best discriminated among bacterial (A) or fungal (B) communities associated with *P*. *penetrans* and *M*. *incognita* after inoculation in soil M10.23 or M10.56.** The OTU extracted by SIMPER analysis contributed to 50% of the Bray-Curtis dissimilarity profile. For each OTU, the species with highest % identity of 16S rRNA genes or fungal ITS in GenBank or UNITE databases are shown.

Fungal OTU detected on the two nematode species after incubation in soil had a higher degree of overlap than bacterial OTU. The nbGLM analysis revealed 91 fungal OTU significantly enriched between the two nematode species (29 on *P*. *penetrans* and 62 on *M*. *incognita*; S2 Table). Among the fungal OTU found in more than one sample, 71% OTU were associated with both species of the nematodes, 11% were specific for *P*. *penetrans*, and 18% were specific for *M*. *incognita*. Among the bacterial OTU found in more than one sample, 63% were shared, 31% were unique to *P*. *penetrans*, and 7% were unique to *M*. *incognita*. The bacterial and fungal OTU that most contributed to the differences in microbial attachment between *M*. *incognita* and *P*. *penetrans* were determined by SIMPER analysis and displayed in heat maps (Fig 2). These discriminating OTU were not found exclusively on one of the nematode species but differed quantitatively. The most discriminating bacteria belonged to Firmicutes, Betaproteobacteria, the Gammaproteobacterium *Acinetobacter johnsonii*, and an unclassified Gemmatimonadetes (Fig 2A). Betaproteobacteria were significantly more abundant on *P*. *penetrans* (*P* = 0.003) especially in soil M10.23. Among these, *Ralstonia insidiosa* and the phytopathogen *Ralstonia solanacearum* were highly abundant on *P*. *penetrans* in soil M10.23 and M10.56. Both species were detected in only one sample of *M*. *incognita*. The genera *Pelomonas*, *Burkholderia* and *Paraburkholderia* were abundant on both nematodes but more frequent on *P*. *penetrans*. The Firmicutes genera *Staphylococcus*, *Streptococcus*, *Bacillus* and *Anaerococcus* were also more frequently detected on *P*. *penetrans*.

The most frequently detected fungal genus was *Malassezia*, with *Malassezia restricta* and *Malassezia* sp. (SH176396.07FU) being more common for *M*. *incognita* (Fig 2B), and *Malassezia globosa* being more frequent on *P*. *penetrans*. *Aspergillus niger* and *Taphrina padi* were highly associated with *P*. *penetrans*, while some OTU belonging to the Dothideomycetes or Sordariomycetes, and the genera *Penicillium* and *Tetracladium* showed a stronger association with *M*. *incognita*.

High-throughput amplicon sequencing did not reveal a significant difference between soils M10.23 and M10.56 in the fungal or bacterial communities associated with *M*. *incognita* or *P*. *penetrans* based on PERMANOVA. However, constrained PCA of these data indicated a difference in the microbiomes of *M*. *incognita* between soils M10.56 and M10.23 (Fig 1), the latter soil being highly suppressive towards root-knot nematodes. Four fungal OTU of the family Plectosphaerellaceae and one OTU assigned to *Malassezia globosa* were much more abundant on *M*. *incognita* from the suppressive soil (Table 2). Only one bacterial OTU, most similar to *Neorhizobium galegae*, was specifically associated with *M*. *incognita* in soil M10.23.

**Table 2:** Fungal and bacterial OTU with higher abundance on *Meloidogyne incognita* in the suppressive soil M10.23 compared to soil M10.56.

Most similar species	% Identity	Accession No.	Family
*Lectera longa*	99.6	SH174362.07FU	Plectosphaerellaceae
*Gibellulopsis nigrescens*	100.0	SH190975.07FU	Plectosphaerellaceae
*Plectosphaerella alismatis*	100.0	SH190976.07FU	Plectosphaerellaceae
*Plectosphaerella cucumerina*	100.0	SH190979.07FU	Plectosphaerellaceae
*Malassezia globosa*	100.0	SH184032.07FU	Malasseziaceae
*Neorhizobium galegae*	99.8	HG938355.1	Rhizobiaceae

OTU which showed the highest difference in average log-transformed relative abundances of soil M1023 compared to soil M10.56 (> 0.8), and which were detected in all samples of *M*. *incognita* from soil M10.23. Other OTU with lower enrichment on *M*. *incognita* but statistically different among soils were listed in S3 Table.

## Discussion

### Root-knot and lesion nematodes were colonized by different specific microbiomes selected from the soil community

Bacterial and fungal community profiling by DGGE and high-throughput amplicon sequencing revealed significant differences in the nematode-associated microbiomes of *Meloidogyne incognita* and *Pratylenchus penetrans* in most of the soils investigated. Attached fungi and bacteria represented a small specific subset of the soil microbiome, when comparing DGGE profiles from total soil DNA and nematodes, with most of the attached species having a minor relative abundance in soil and being strongly enriched on the nematode surface. This could imply a nematode-selective recruitment of soil microbes. The associated microbiomes were highly determined by nematode species as several genera were more abundant on *P*. *penetrans*.

With respect to relatively low microbial abundance on both nematodes in the two soils tested by NGS and strong influence of nematode species on the attachment, we presume that surface epitopes of the surface coat between the two nematode species differ. Davies and Curtis [13] explained the importance of the surface coat in nematode-plant-microbe interactions. The surface coat is a highly glycosylated lipoprotein layer that covers the cuticle of nematodes. Its importance in nematode-microbe interactions was studied on several occasions in the past. Spiegel et al. [40] observed the binding of human red blood cells to juveniles of *Meloidogyne javanica* and *Pratylenchus mediterraneus* and some other phytonematodes. They suggested that carbohydrate-recognition domains in the surface coat were involved in the specific binding as removal of the surface coat by detergent or treatment with carbohydrates or trypsin decreased binding, and it was restored within 24 hours after renewal of the surface coat. Similarly, the attachment of coryneform bacteria (*Clavibacter* sp.) to dauer juveniles of *Anguina agrostis* increased 18 hours after hydrolysis of the nematode surface coat glycoproteins [41]. Nevertheless, a selectivity and specificity of a nematode-microbe relationship seems to be much more complex as in the case of the gram-positive obligate parasite *Pasteuria penetrans* whose binding is specific for different host populations [42]. Therefore, it was expected to have a significant difference in attached microbiomes between the two nematode species in our study. As discussed by Davies and Danks [15] the amount of surface coat associated proteins differs between nematode species and this may play a role in the recognition of the nematode by soil microbes. Also, it should be pointed out that infective stages of *M*. *incognita* are only J2, while in case of *P*. *penetrans* all life stages are infective. This could influence our results as the surfaces of life stages differ [40] and might attract different microbial species [43, 44].

*Malassezia*, *Aspergillus* and *Cladosporium* were the most frequent fungal genera detected on both nematodes, with *Malassezia restricta* being more common for *M*. *incognita*, and *Malassezia globosa* or *Malassezia* sp. for *P*. *penetrans*. *M*. *restricta* was also detected attached to the cuticle of *M*. *hapla* J2 when exposed to three soils showing different levels of suppression to the root-knot nematode [16]. Moreover, *M*. *restricta* and *M*. *globosa* were previously reported associated to the plant ectoparasitic nematode *Malenchus* sp. [45]. *Malassezia* was often found on the skin of mammals, sometimes causing skin disease [46], and attachment to the cuticle of the nematodes might be based on molecular structures shared with vertebrates [47].

Betaproteobacteria, Bacilli and Actinobacteria were the most frequent bacterial classes detected on both nematodes. In our study we aimed to completely remove loosely attached microbes from the nematodes by several washing steps. In contrast, Cao et al. [43] analyzed the bacteria associated with J2 of *M*. *incognita* without such effort and found a diversity of bacterial genera which much resembled what is typically found in soil.

### Soil type influenced nematode-associated microbiomes

In DGGE fingerprints the total microbial communities of soils conducted under organic production clustered separately from soils under integrated management, except soil M10.16, while the nematode-recruited fractions were unrelated. This showed that the majority of abundant soil microbial species, which determined the fingerprints, did not attach to the nematodes but that less abundant species were associated with the two nematodes. High-throughput amplicon sequencing of the fungi and bacteria associated with the nematodes did not reveal significant differences among the two soils which were selected based on their similarity in total microbial community structure and their observed difference in suppressiveness towards root-knot nematodes. However, some fungal species of the family Plectosphaerellaceae, *Malassezia globosa* and the rhizobacterium *Neorhizobium galegae* were much more frequently detected on *M*. *incognita* from the suppressive soil, but this was not statistically significant due to variation between replicates. When expanding the study to seven more dissimilar soils using DGGE, the soil type showed over all a significant influence on bacterial and fungal attachment to both of the nematodes. This soil effect was rather small compared to the effect of the nematode species on microbial attachment (Table 1).

### Putative ecological role of attached microbes

A high selectivity and specificity has allowed the attachment of a small subset of the myriad of soil microorganisms to nematodes tested in our study. This non-random attachment of microbial species to one or both of the investigated nematodes suggested a particular ecological role for these associations. We did not find the well-known genera parasitizing nematodes or eggs of nematodes, like *Pasteuria*, *Pochonia* or *Paecilomyces* [3–10]. However, enrichment of *Burkholderia* spp. on both nematode species was observed. This genus was positively correlated with *Pratylenchus zeae* [48]. Many *Burkholderia* species are typical root endophytes [49], some are able to colonize egg masses [50], and to paralyze J2 of root-knot nematodes [51]. *Penicillium chrysogenum*, *Aspergillus niger* or some growth promoting bacteria suppressed root-knot nematode reproduction on tomato when used with cattle manure [52]. In addition, the dry mycelium of *Penicillium chrysogenum* reduced root galling on cucumber and tomato, while the water extract only paralyzed J2 and reduced egg hatching in a reversible manner [53]. *Chaetomium* sp., and *Plectosphaerella cucumerina* can parasitize eggs or be antagonists of root-knot nematodes [10, 54, 55, 56]. In our study, the attached microbiomes did not statistically differ between the highly suppressive soil and a comparable soil, which was not conspicuously suppressive towards root-knot nematodes. However, *Malassezia globosa* and four fungal species of the family Plectosphaerellaceae, and the bacterium *Neorhizobium galegae* were strongly enriched on *M*. *incognita* in the suppressive soil.

The nematodes may also carry plant-beneficial microbes to the plant. Species of the fungal genus *Gibellulopsis* were among the attached fungi. *Gibellulopsis nigrescens* is a low pathogenic fungus of mentha, tomato, pepper and eggplant, which can control virulent isolates of *Verticillium dahliae* [57, 58]. The interactions of the attached microbial species with crop plants still need to be investigated. Endosymbiotic genera like *Wolbachia*, as previously studied in *P*. *penetrans* [59], were not detected in this study.

Endoparasitic phytonematodes may play a role in carrying phytopathogenic bacteria and fungi to plants, open a wound to facilitate their entrance [60], or carry them actively into the root. Plant disease complexes of soilborne pathogens and phytonematodes were often reported, but attachment to infective stages of endoparasitic nematodes were not considered as a mechanism underlying such disease complexes [61]. *Ralstonia solanacearum*, a pathogen of a wide range of plants including vegetables [62], was found in our study attached to *P*. *penetrans* but not to *M*. *incognita*. Root-knot nematodes have been reported to enhance the disease of this wilting bacterium on binjal [63]. The fungus *Taphrina padi*, a pathogen of *Prunus padus* [64], was identified on *P*. *penetrans*. The fungus *Alternaria alternata*, being also a pathogen of vegetables [62], was identified on *M*. *incognita*.

Several human-associated bacteria and fungi were detected on both *M*. *incognita* and *P*. *penetrans*, such as aforementioned *M*. *restricta* and *M*. *globosa*. These fungi were previously detected in association with some free-living nematodes [45], but were most probably ingested. However, plant-parasitic nematodes do not feed on any other organisms but plants and can only allow microbes to attach to their surface. The possibility of plant-parasitic nematodes to transmit pathogens of humans has been poorly investigated. The adhesion of *Escherichia coli* to *M*. *javanica* was reported [65], but transmission to the plant has not been explored. However, the wounding of spinach roots with *M*. *hapla* did not enable entry of *E*. *coli* to the roots [66], suggesting no role of root-knot nematodes in transmission of this bacterium.

## Concluding remarks

The highly specific attachment of bacterial and fungal species from soil to infective stages of endoparasitic nematodes suggests that these microbes may have a specific ecological function. As the microbes attach to the nematode just before they invade the plant root, it was interesting to investigate whether they induce plant defense and thereby the specific rhizosphere community might lead to a protection of the plant. The different host plants of root-knot and lesion nematodes might then be a clue to understand the observed differences in attached microbial species of these two nematodes. This needs to be investigated and might be the basis for new control strategies against phytonematodes.

## Supporting information

S1 Fig Fungal communities associated with *P. penetrans* or *M. incognita* in seven horticultural soils. Fungal ITS1 fragments were amplified from total DNA of clean nematodes before and after incubation in soil suspensions and separated in DGGE.
(PDF)

S2 Fig Bacterial communities associated with *P. penetrans* or *M. incognita* in seven horticultural soils. Bacterial 16S rRNA gene fragments were amplified from total DNA of clean nematodes before and after incubation in soil suspensions and separated in DGGE.
(PDF)

S3 Fig Fungal communities of the seven horticultural soils analyzed. Fungal ITS1 fragments were amplified from total DNA of 0.5 gram soil and separated in DGGE. Differences between the fungal soil fingerprints were statistically tested by a permutation test based on their pairwise Pearson correlations within and between soils. The back box shows the global P-value (P = 0 means P<0.001), and the corrected pairwise P-values (group 1 corresponds to soil M10.16; group 2 corresponds to M10.55; etc.) of this permutation test as described by Kropf et al.
(PDF)

S4 Fig Bacterial communities of the seven horticultural soils analyzed. 16S rRNA fragments were amplified from total DNA of 0.5 gram soil and separated in DGGE. Differences between the bacterial soil fingerprints were statistically tested by a permutation test based on their pairwise Pearson correlations within and between soils. The back box shows the global P-value (P = 0 means P<0.001), and the corrected pairwise P-values (group 1 corresponds to soil M10.16; group 2 corresponds to M10.55; etc.) of this permutation test as described by Kropf et al.
(PDF)

S1 Table Physicochemical properties and enzymatic activities of the soils in this study. (PDF)

S2 Table Dominant fungal species significantly different between the two nematode species regardless of the soils (average ± SEM). Only species with a relative abundance > 0.1% are displayed. Significance was inferred using a negative binomial regression and generalized linear model fit (nbGLM). A Likelihood ratio test was used, complemented with a post-hoc false discovery rate multiple correction test (LHR FDR, *P* < 0.05). Analysis was done using the EdgeR package.
(PDF)

S3 Table Fungal OTU associated with *Meloidogyne incognita* with significantly different abundance between the soils M10.23 and M10.56 (average ± SEM). Significance was inferred using a negative binomial regression and generalized linear model fit (nbGLM). A Likelihood ratio test was used, complemented with a post-hoc false discovery rate multiple correction test (LHR FDR, *P*< 0.05). Analysis was done using the EdgeR package.
(PDF)
