# Supplementary material for: Microbiomes associated with infective stages of root-knot and lesion nematodes in soil
Source: PLoS One. 2017 May 4;12(5):e0177145. doi: 10.1371/journal.pone.0177145 (PMC5417685; doi:10.1371/journal.pone.0177145)
Supplement: S1 Fig — Fungal ITS1 fragments were amplified from total DNA of clean nematodes before and after incubation in soil suspensions and separated in DGGE. (PDF) [file pone.0177145.s001.pdf]

S1 Fig. Fungal communities associated with *P. penetrans* or *M. incognita* in seven horticultural soils. Fungal ITS1 fragments were amplified from total DNA of clean nematodes before and after incubation in soil suspensions and separated in DGGE.

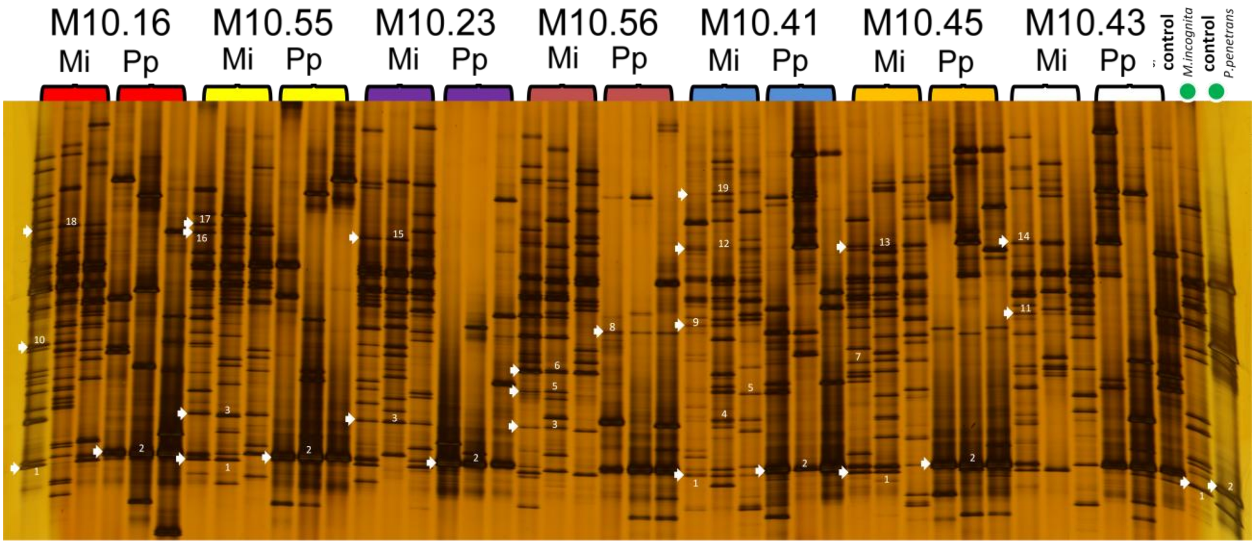

Bands no. 3 and 5 were specific for *M. incognita* in two or three of all studied soils, respectively. Bands no. 6 - 19 were specifically associated with only one soil. Only a few soils had a significant influence on attached fungi, with soils M10.56 and M10.43 especially differing to others (soils M10.23 vs. M10.43, and M10.56 vs. M10.43 for *M. incognita*; soils M10.56 vs. M10.43 and M10.56 vs. M10.45 for *P. penetrans*).
