# Supplementary material for: Microbiomes associated with infective stages of root-knot and lesion nematodes in soil
Source: PLoS One. 2017 May 4;12(5):e0177145. doi: 10.1371/journal.pone.0177145 (PMC5417685; doi:10.1371/journal.pone.0177145)
Supplement: S2 Fig — Bacterial 16S rRNA gene fragments were amplified from total DNA of clean nematodes before and after incubation in soil suspensions and separated in DGGE. (PDF) [file pone.0177145.s002.pdf]

S2 Fig. Bacterial communities associated with *P. penetrans* or *M. incognita* in seven horticultural soils. Bacterial 16S rRNA gene fragments were amplified from total DNA of clean nematodes before and after incubation in soil suspensions and separated in DGGE.

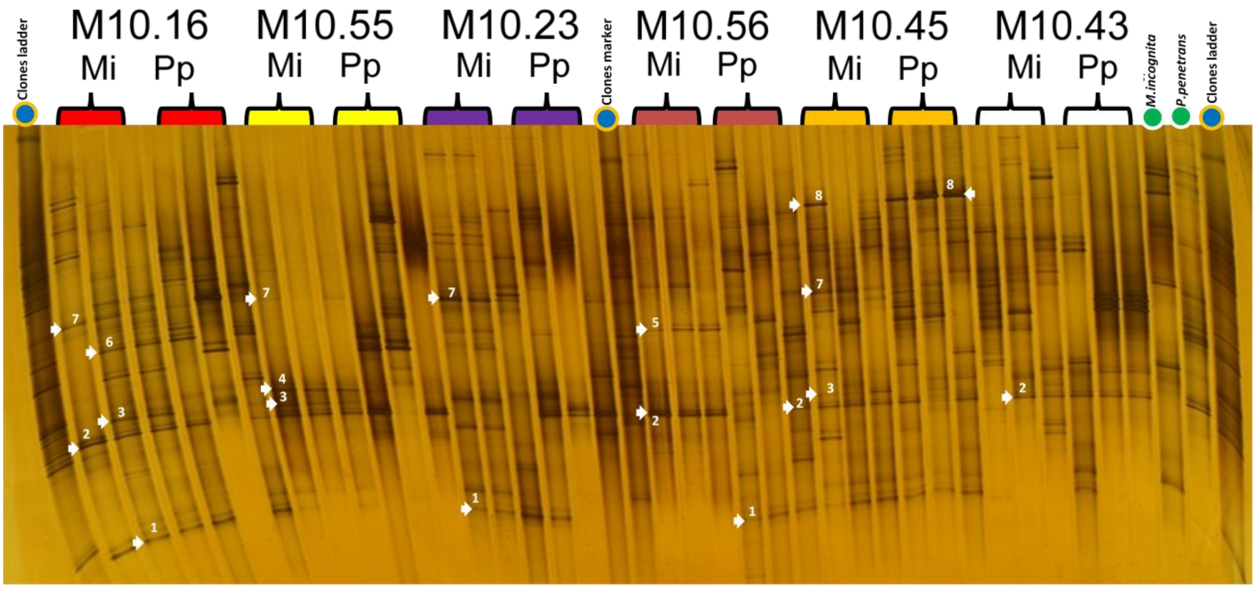

Sequences of 16S rRNA of bacteria attached to the phytonematodes *M. incognita* and *P. penetrans* corresponding to DGGE bands:

Band no.	Most similar bacterial species	Identity %	Accession no.
1	*Burkholderia ginsengisoli*	95%	KY432386
2	*Fusicatenibacter saccharivorans*	99.5%	KY432387
3	*Burkholderia lata*	97%	KY432394
4	*Oscillatoria nigro-viridis*	99.3%	No Accession no.
5	*Burkholderia rhizoxinica*	97%	KY432388
6	*Burkholderia caledonica*	98%	KY432390
7	*Curvibacter sp*	91%	No Accession no.
8	*Acinetobacter johnsonii*	98.9%	KY432396

Band no. 7 (91% sequence identity to *Curvibacter* sp.) appeared in four soils (M10.16, M10.55, M10.23 and M10.45).
