# Supplementary material for: Microbiomes associated with infective stages of root-knot and lesion nematodes in soil
Source: PLoS One. 2017 May 4;12(5):e0177145. doi: 10.1371/journal.pone.0177145 (PMC5417685; doi:10.1371/journal.pone.0177145)
Supplement: S3 Fig — Fungal ITS1 fragments were amplified from total DNA of 0.5 gram soil and separated in DGGE. Differences between the fungal soil fingerprints were statistically tested by a permutation test based on their pairwise Pearson correlations within and between soils. The back box shows the global P-value (P = 0 means P<0.001), and the corrected pairwise P-values (group 1 corresponds to soil M10.16; group 2 corresponds to M10.55; etc.) of this permutation test as described by Kropf et al. (PDF) [file pone.0177145.s003.pdf]

S3 Fig. **Fungal communities of the seven horticultural soils analyzed.** Fungal ITS1 fragments were amplified from total DNA of 0.5 gram soil and separated in DGGE. Differences between the fungal soil fingerprints were statistically tested by a permutation test based on their pairwise Pearson correlations within and between soils. The back box shows the global P-value (P=0 means P<0.001), and the corrected pairwise P-values (group 1 corresponds to soil M10.16; group 2 corresponds to M10.55; etc.) of this permutation test as described by Kropf et al. [1].

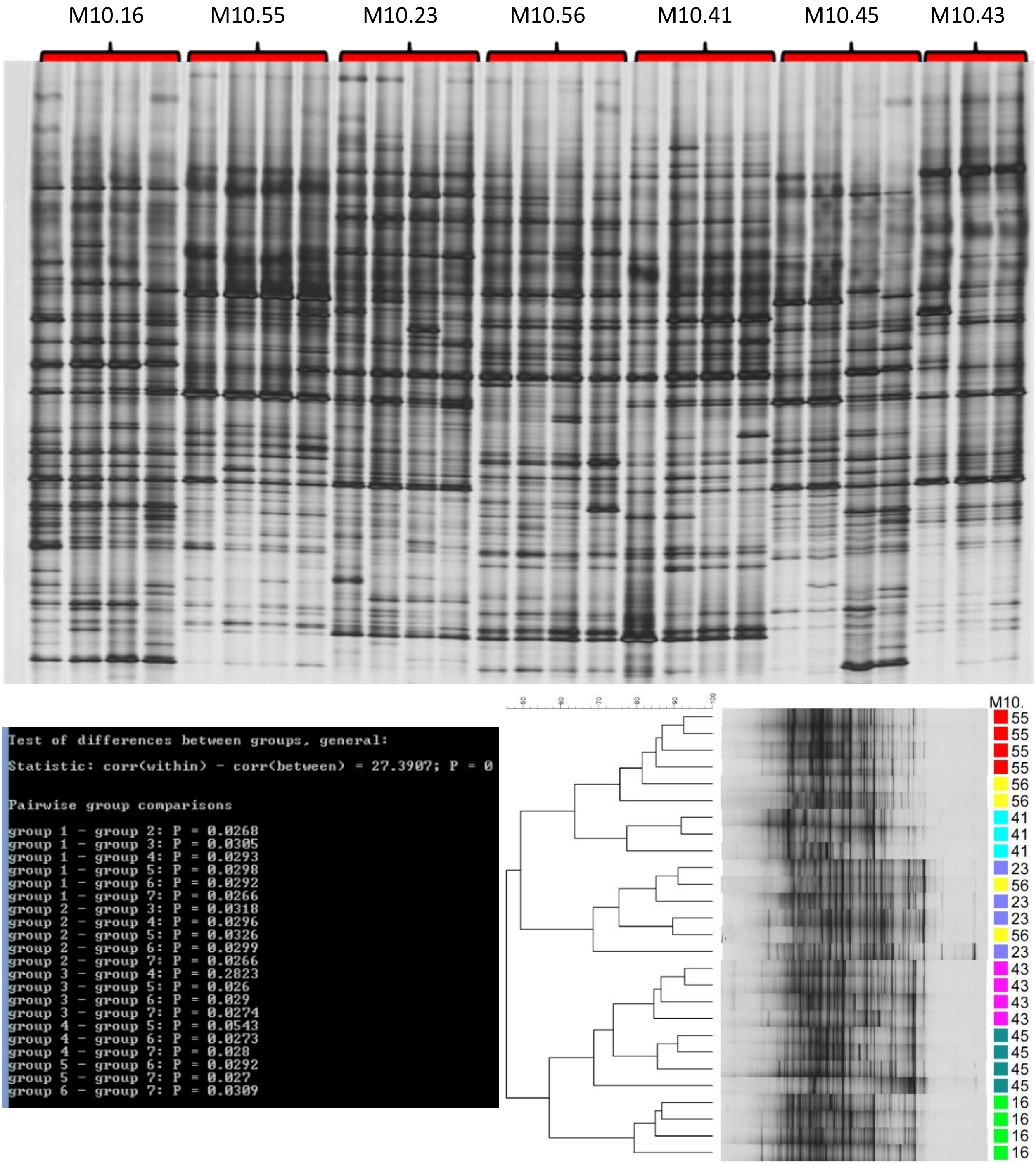

1. Kropf S, Heuer H, Grüning M, Smalla K. Significance test for comparing complex microbial community fingerprints using pairwise similarity measures. J Microbiol Methods. 2004; 57: 187-95. doi: 10.1016/j.mimet.2004.01.002.
