# Supplementary figures and images for: Microbiomes associated with infective stages of root-knot and lesion nematodes in soil
Source: PLoS One. 2017 May 4;12(5):e0177145. doi: 10.1371/journal.pone.0177145 (PMC5417685; doi:10.1371/journal.pone.0177145)

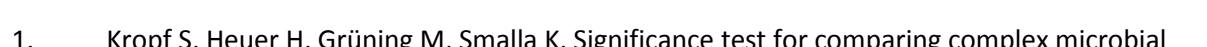

Supplement: S4 Fig — 16S rRNA fragments were amplified from total DNA of 0.5 gram soil and separated in DGGE. Differences between the bacterial soil fingerprints were statistically tested by a permutation test based on their pairwise Pearson correlations within and between soils. The back box shows the global P-value (P = 0 means P<0.001), and the corrected pairwise P-values (group 1 corresponds to soil M10.16; group 2 corresponds to M10.55; etc.) of this permutation test as described by Kropf et al. (PDF) [file pone.0177145.s004.pdf]
