# Supplementary material for: Microbiomes associated with infective stages of root-knot and lesion nematodes in soil
Source: PLoS One. 2017 May 4;12(5):e0177145. doi: 10.1371/journal.pone.0177145 (PMC5417685; doi:10.1371/journal.pone.0177145)
Supplement: S1 Table — (PDF) [file pone.0177145.s005.pdf]

S1 Table. Physicochemical properties and enzymatic activities of the soils in this study.

Variable	Soil						
	M10.16	M10.23	M10.41	M10.43	M10.45	M10.55	M10.56
Geografic location N	40.413°	40.677°	40.676°	40.518°	40.518°	40.542°	40.542°
Geografic location E	1.5155°	0.5772°	0.5772°	2.4074°	2.4126°	0.4983°	0.4977°
Horticultural production	organic	organic	organic	integrated	integrated	organic	organic
Sand (%)	50	45	33	75	67	68	53
Silt (%)	30	40	38	15	14	0	29
Clay (%)	20	15	29	10	19	32	18
Soil texture (USDA)	Loam	Loam	Clay loam	Sandy loam	Sandy loam	Sandy clay loam	Sandy loam
pH (1:2.5 in water)	8.25	8.3	8.17	8.13	7.77	8.1	8.32
Organic matter (w/w)	1.2	5.77	4.38	1.51	2.5	2.5	4.29
Electric conductivity (µS/cm) (1:5)	300	276	516	155.6	332	1069	415
B (ppm)	2.62	2.8	5.33	2.82	0.75	1.12	1.21
Exchangeable Ca (meq 100 g^{-1})	14.15	17.32	14.88	7.18	9.98	18.16	15.89
Available Ca (meq 100 g^{-1})	15.74	17.16	14.7	8.14	10.74	18.99	16.96
$CaCO_3$ (%)	6	3.8	4	1	3.8	4.1	5.1
Cation exchange capacity (meq 100 g^{-1})	26.84	41.17	14.06	9.12	12.22	25.7	22.89
Cu (ppm)	2.5	3.62	2.5	28.31	28.77	2.5	2.5
Available P (ppm)	118.25	379.4	247.47	57.94	86.92	75.79	80.19
Fe (ppm)	5	11.37	5	239.07	79.72	5	5
Exchangeable Mg (meq 100 g^{-1})	1.37	3.99	4.8	0.91	1.41	2.97	2.21
Available Mg (meq 100 g^{-1})	1.43	4.97	4.43	1.06	1.58	3.66	2.72
Mn (ppm)	55.54	63.92	92.44	92.23	54.26	2.5	2.5
N (ppm)	522.9	2329	2388.7	629.8	1824.9	1497.7	2209.8
Exchangeable K (meq 100 g^{-1})	0.59	1.19	1.47	0.15	0.35	0.67	0.62
Available K (meq 100 g^{-1})	0.79	1.89	1.67	0.18	0.43	0.69	1.05
C/N	13.36	14.38	10.64	13.87	7.94	9.68	11.26
Exchangeable Na (meq 100 g^{-1})	0.46	0.29	0.57	0.34	0.36	0.54	0.37
Available Na (meq 100 g^{-1})	0.38	1.02	1.85	0.36	0.54	3.17	0.62
Zn (ppm)	2.64	20.61	7.19	24.63	24.52	2.5	2.5
Ca + Mg/K	26.31	17.91	13.39	53.93	32.54	31.54	29.20
P/N	0.23	0.16	0.10	0.09	0.05	0.05	0.04
Fluorescein diacetate hydrolysis (µg fluorescein h^{-1} x g soil)	2.85	5.45	4.44	2.79	3.58	0.95	3.86
β-glucosaminidase (µmols *p*-nitrophenol h^{-1} x g soil)	0.11	0.40	0.30	0.20	0.33	0.10	0.42
Urease (µmols N–NH_4 h^{-1} x g soil)	0.57	1.58	2.24	0.57	1.21	0.87	1.34
Protease (µg tyrosine h^{-1} x g soil)	5.02	4.49	5.42	8.89	9.49	12.42	8.79
