# Supplementary material for: Microbiomes associated with infective stages of root-knot and lesion nematodes in soil
Source: PLoS One. 2017 May 4;12(5):e0177145. doi: 10.1371/journal.pone.0177145 (PMC5417685; doi:10.1371/journal.pone.0177145)
Supplement: S2 Table — Only species with a relative abundance > 0.1% are displayed. Significance was inferred using a negative binomial regression and generalized linear model fit (nbGLM). A Likelihood ratio test was used, complemented with a post-hoc false discovery rate multiple correction test (LHR FDR, P < 0.05). Analysis was done using the EdgeR package. (PDF) [file pone.0177145.s006.pdf]

S2 Table. Dominant fungal species significantly different between the two nematode species regardless of the soils (average ± SEM). Only species with a relative abundance > 0.1% are displayed. Significance was inferred using a negative binomial regression and generalized linear model fit (nbGLM). A Likelihood ratio test was used, complemented with a post-hoc false discovery rate multiple correction test (LHR FDR, *P* < 0.05). Analysis was done using the EdgeR package.

Phylum	Family	Species	*Pratylench*	*Meloidogy*	Adj. FDR
Ascomycota	Ascomycota (unid.)	SH212862.07FU	-	0.49±0.49b	3.91E-05
	Ascomycota (unid.)	SH210351.07FU	-	0.49±0.47b	1.77E-04
	Chaetomiaceae	*Chaetomium jatrophae*	-	1.37±1.25b	9.27E-05
	Chaetomiaceae	*Humicola grisea*	-	0.13±0.11b	2.04E-03
	Chaetomiaceae	*Chaetomium aterrimum*	-	0.3±0.3b	4.17E-03
	Cordycipitaceae	*Cordyceps bassiana*	-	0.3±0.3b	6.64E-03
	Davidiellaceae	*Cladosporium exasperatum*	0.03±0.01a	9.16±3.01b	1.77E-04
	Dothideomycetes	unidentified	-	1.15±1.15b	7.14E-05
	Dothioraceae	*Aureobasidium pullulans*	-	1.91±1.25b	5.59E-05
	Helotiales (i.sedis)	*Tetracladium maxilliforme*	0.01±0.01a	5.2±4.57b	7.14E-05
	Helotiales (i.sedis)	unidentified	-	0.92±0.91b	3.41E-03
	Hyaloscyphaceae	*Mycoarthris corallina*	-	0.48±0.47b	5.05E-05
	Hypocreales (i.sedis)	*Myrothecium roridum*	-	0.11±0.11b	0.013
	Mycosphaerellaceae	*Pseudocercosporella*	-	0.41±0.41b	5.05E-05
	Nectriaceae	SH219102.07FU	0.01±0.002	1.9±1.89b	9.27E-05
	Nectriaceae	SH220702.07FU	0.02±0.01a	6.15±3.52b	2.12E-04
	Onygenaceae	*Chrysosporium lobatum*	-	0.31±0.31b	5.55E-03
	Pezizales (i.sedis)	*Cephaliophora tropica*	-	0.63±0.63b	3.17E-03
	Plectosphaerellaceae	*Lectera longa*	-	1.54±1b	3.82E-05
	Plectosphaerellaceae	*Plectosphaerella alismatis*	-	0.18±0.11b	3.61E-03
	Pleosporaceae	*Stemphylium herbarum*	-	5.08±2.6b	3.91E-05
	Pleosporaceae	*Alternaria alternata*	0.02±0.01a	10.95±7.36	6.20E-05
	Pleosporaceae	*Chalastospora ellipsoidea*	-	0.38±0.33b	1.77E-04
	Pleosporales (unid.)	unidentified	-	0.9±0.9b	3.61E-03
	Pyronemataceae	unidentified	-	0.49±0.49b	3.61E-03
	Pyronemataceae	*Pseudaleuria* sp.	-	0.39±0.39b	5.55E-03
	Saccharomycetales	*Candida tropicalis*	0.01±0.01a	3.65±3.64b	7.14E-05
	Sordariales (unid.)	unidentified.	-	1.3±0.83b	1.00E-04
	Sordariomycetes	unidentified	0.01±0.003	0.75±0.52b	4.17E-03
	Trichocomaceae	*Penicillium chrysogenum*	-	3.51±2.31b	4.57E-05
	Trichocomaceae	*Penicillium aurantiogriseum*	-	2.14±2.09b	7.34E-05
	Sordariomycetes	unidentified	0.01±0.003	4.59±2.63b	2.12E-04
Basidiomycota	Tremellales (i.sedis)	*Cryptococcus heimaeyensis*	-	2.75±2.74b	1.54E-05
	Tremellales (i.sedis)	*Hannaella oryzae*	-	3.47±3.47b	5.05E-05
Chytridiomycota	Rhizophlyctidaceae	*Rhizophlyctis rosea*	-	0.12±0.12b	0.012
	Rhizophydiaceae	*Rhizophydium* sp.	-	0.18±0.18b	6.64E-03
Fungi (unid.)	Fungi (unid.)	unidentified	-	0.3±0.3b	5.62E-05
Ascomycota	Davidiellaceae	*Cladosporium haerospermum*	1.04±1.04b	-	3.82E-05
	Davidiellaceae	*Cladosporium halotolerans*	1.54±1.53b	-	1.51E-03
	Hypocreaceae	*Trichoderma asperellum*	0.96±0.96b	-	5.50E-05
	Hypocreales (i.sedis)	unidentified	0.47±0.47b	-	3.91E-05
	Saccharomycetales	*Debaryomyces nepalensis*	0.9±0.9b	-	3.91E-05
	Taphrinaceae	*Taphrina padi*	0.65±0.49b	-	3.82E-05
	Trichocomaceae	*Penicillium brevicompactum*	0.21±0.21b	-	5.44E-03
	Trichocomaceae	*Aspergillus niger*	63.35±4.76	1.77±1.72a	8.49E-03
	Trichocomaceae	*Penicillium novae-zeelandiae*	0.12±0.12b	0.01±0.01a	0.037
	unidentified	*Pleosporales* sp.	2.12±2.11b	0.01±0a	2.96E-04
	Xylariaceae	*Hypoxylon howeanum*	0.37±0.37b	-	5.62E-05
Basidiomycota	Malasseziaceae	*Malassezia* SH176396.07FU	2.77±2.61b	-	5.47E-05
	Malasseziaceae	*Malassezia* SH176395.07FU	0.31±0.31b	-	4.17E-03
	Marasmiaceae	*Baeospora myosura*	1.7±1.69b	-	1.07E-03
Zygomycota	Mortierellaceae	*Mortierella alpina*	1.47±1.46b	-	4.57E-05
