# Supplementary material for: Microbiomes associated with infective stages of root-knot and lesion nematodes in soil
Source: PLoS One. 2017 May 4;12(5):e0177145. doi: 10.1371/journal.pone.0177145 (PMC5417685; doi:10.1371/journal.pone.0177145)
Supplement: S3 Table — Significance was inferred using a negative binomial regression and generalized linear model fit (nbGLM). A Likelihood ratio test was used, complemented with a post-hoc false discovery rate multiple correction test (LHR FDR, P< 0.05). Analysis was done using the EdgeR package. (PDF) [file pone.0177145.s007.pdf]

S3 Table. Fungal OTU associated with *Meloidogyne incognita* with significantly different abundance between the soils M10.23 and M10.56 (average ± SEM). Significance was inferred using a negative binomial regression and generalized linear model fit (nbGLM). A Likelihood ratio test was used, complemented with a post-hoc false discovery rate multiple correction test (LHR FDR, *P*< 0.05). Analysis was done using the EdgeR package.

Phylum	Class	Species	OTU	Soil M10.23	Soil M10.56	FDR *P*
Ascomycota	Chaetomiaceae	*Chaetomium aterrimum*	SH204872.07FU	0	0.599 ±0.599	0.022
Ascomycota	Incertae sedis	*Candida tropicalis*	SH201993.07FU	0.015 ±0.009	7.287 ±7.284	0.024
Ascomycota	Pyronemataceae	unidentified	SH213756.07FU	0.001 ±0.001	0.984 ±0.984	0.022
Ascomycota	Trichocomaceae	*Penicillium aurantiogriseum*	SH407694.07FU	0.004 ±0.002	4.269 ±4.159	0.017
Ascomycota	unidentified	unidentified	SH210351.07FU	0.005 ±0.004	0.978 ±0.929	0.024
Ascomycota	unidentified	unidentified	SH212862.07FU	0.002 ±0.002	0.984 ±0.984	0.021
Ascomycota	unidentified	*Dothideomycetes* sp.	SH213019.07FU	0.002 ±0.001	2.297 ±2.297	0.017
Basidiomycota	Incertae sedis	*Hannaella oryzae*	SH219461.07FU	0.003 ±0.001	6.931 ±6.931	0.017
Ascomycota	Incertae sedis	*Cephaliophora tropica*	SH217807.07FU	1.25 ±1.25	0.001 ±0.001	0.022
Ascomycota	Nectriaceae	unidentified	SH219102.07FU	3.80 ±3.78	0.003 ±0.001	0.029
Ascomycota	unidentified	*Pleosporales* sp.	SH206635.07FU	1.79 ±1.79	0.001 ±0.001	0.017
Basidiomycota	Incertae sedis	*Cryptococcus heimaeyensis*	SH181631.07FU	5.49 ±5.47	0.001 ±0.001	0.017
Chytridiomycota	Rhizophydiaceae	*Rhizophydium* sp.	SH210678.07FU	0.35 ±0.35	0.001 ±0.001	0.029
unidentified	unidentified	*Fungi* sp.	SH195781.07FU	0.60 ±0.60	0	0.024
